# The Drosophila anatomy ontology

**DOI:** 10.1186/2041-1480-4-32

**Published:** 2013-10-18

**Authors:** Marta Costa, Simon Reeve, Gary Grumbling, David Osumi-Sutherland

**Affiliations:** 1 FlyBase, Department of Genetics, University of Cambridge, Downing Street, Cambridge, UK; 2 FlyBase, Department of Biology, Indiana University, 1001 E 3rd Street, Bloomington, IN, 47405-7005, USA

**Keywords:** Drosophila, Anatomy, Ontology, OWL, OBO, Gene Ontology, FlyBase, Description logic

## Abstract

**Background:**

Anatomy ontologies are query-able classifications of anatomical structures. They provide a widely-used means for standardising the annotation of phenotypes and expression in both human-readable and programmatically accessible forms. They are also frequently used to group annotations in biologically meaningful ways. Accurate annotation requires clear textual definitions for terms, ideally accompanied by images. Accurate grouping and fruitful programmatic usage requires high-quality formal definitions that can be used to automate classification and check for errors. The *Drosophila* anatomy ontology (DAO) consists of over 8000 classes with broad coverage of *Drosophila* anatomy. It has been used extensively for annotation by a range of resources, but until recently it was poorly formalised and had few textual definitions.

**Results:**

We have transformed the DAO into an ontology rich in formal and textual definitions in which the majority of classifications are automated and extensive error checking ensures quality. Here we present an overview of the content of the DAO, the patterns used in its formalisation, and the various uses it has been put to.

**Conclusions:**

As a result of the work described here, the DAO provides a high-quality, queryable reference for the wild-type anatomy of *Drosophila melanogaster* and a set of terms to annotate data related to that anatomy. Extensive, well referenced textual definitions make it both a reliable and useful reference and ensure accurate use in annotation. Wide use of formal axioms allows a large proportion of classification to be automated and the use of consistency checking to eliminate errors. This increased formalisation has resulted in significant improvements to the completeness and accuracy of classification. The broad use of both formal and informal definitions make further development of the ontology sustainable and scalable. The patterns of formalisation used in the DAO are likely to be useful to developers of other anatomy ontologies.

## Background

### Anatomy ontologies

Anatomy ontologies are queryable classifications of anatomical structures. They are commonly used by bioinformatics resources to provide controlled vocabularies for annotating a range of entities (such as research papers, genes and genotypes). Typically curation is done manually and consists of assertions about phenotypes and expression patterns [1-4] but many other types of assertion are possible. For manual annotation, class and part hierarchies in ontologies provide terms with a range of specificity allowing curators to choose an appropriately precise term depending on the information available. Term names alone are frequently ambiguous. Textual definitions of terms, ideally supplemented with images, are therefore important for consistent and accurate manual annotation.

Anatomy ontologies are also used to group annotations in biologically meaningful ways. This is commonly done by grouping annotations using class and part hierarchies (partonomy). For example, a query for genes expressed in the *Drosophila* leg would return gene expression annotated with the term middle leg (a subclass of leg) and claw (a part of the leg) as well as with the term leg. The usefulness of such grouping depends on the accuracy of classification and of assertions about partonomy. More sophisticated groupings can be achieved by taking advantage of ontology semantics expressed in a formal language such as OWL. For example, Virtual Fly Brain (VFB) groups annotations based on inference of overlap between neurons and gross neuro-anatomical structures as well as using partonomy and classification [5,6].

Various resources, including model organism databases, use anatomy ontologies as searchable stores of information about anatomy. Annotation of terms with synonyms provides a means for users to search for anatomical structures using the various names used for them in the literature. Textual definitions provide human readable information about anatomy and links to references and in some cases, images. Formal relationships provide a means to browse to related terms as well as for grouping annotations. Formalisation of anatomy ontologies in OWL also has great potential as a source of queryable information about anatomy. For example, VFB uses OWL queries to provide answers to user queries about neuronal connectivity in the *Drosophila* nervous system.

#### Anatomy ontologies and formal ontology languages

OWL 2 Web Ontology Language (OWL2) [7] is a W3C recommended, description-logic based ontology language. Its rigorous definition, web integration and the wide availability of fast reasoners make it a very attractive language to use for ontology building. The EL profile of OWL2 [8] is particularly attractive as reasoning times scale very well with increasing size and complexity and new, fast reasoners that take advantage of this are available [9].

Most widely used anatomy ontologies, or ontologies with major anatomical components such as Snomed-CT (http://www.ihtsdo.org/snomed-ct), were developed prior to the publication of the OWL2 specification and so use different formalisms. One of the most commonly used ontology languages apart from OWL is Open Biomedical Ontologies format (OBO format). Historically, OBO format ontologies have been manually maintained and only weakly formalised compared to what is possible in OWL2. Improvements to the expressiveness of OBO format and the definition of OBO format semantics via mapping to OWL2 [10,11] have made it possible to formalise definitions so that OWL2 reasoners can be used to automate classification, check for consistency and run queries. Referencing terms from external ontologies in formal definitions makes cross ontology querying possible and, when combined with modularisation strategies, allows auto-classification to leverage the semantics of other ontologies. By using OWLtools (https://code.google.com/p/owltools/), it possible to do this while keeping the master version of an ontology in OBO 1.4.

This approach is already being used to improve the Gene Ontology (GO) [12,13], a number of phenotype ontologies [14-16], the Cell Ontology (CL) [17] and is central to construction of the multi-species anatomy ontology Uberon [18]. The expressiveness of OBO1.4 is almost entirely within the EL profile of OWL2, meaning that, with a few precautions, it is possible to take advantage of the new generation of fast EL reasoners when working with OWL2 translations of OBO ontologies.

### A brief history of the *Drosophila* anatomy ontology

The DAO has its origins in a semi-structured, controlled vocabulary developed by Michael Ashburner for use in annotation by FlyBase over 20 years ago. An early draft (https://sourceforge.net/p/fbbtdv/code/HEAD/tree/fbbt/releases/prehistory/proto-FBbt-1992.txt) had over 2500 terms arranged in a single inheritance hierarchy with 76 references, each attached to a mid-level node in the hierarchy. It had no named relations or textual definitions, but its simple structure made it easy for users and editors to follow. Following its initial drafting, the ontology grew largely by addition of terms requested by curators and gradually became more formal with the adoption of standards developed for the GO, including OBO format. With increased formalisation came the use of explicit classification extending to multiple axes (each term could have more than one parent class), and the use of named, although undefined, relations for partonomy and development. During this growth, very few textual definitions were added.

**Figure 1:**
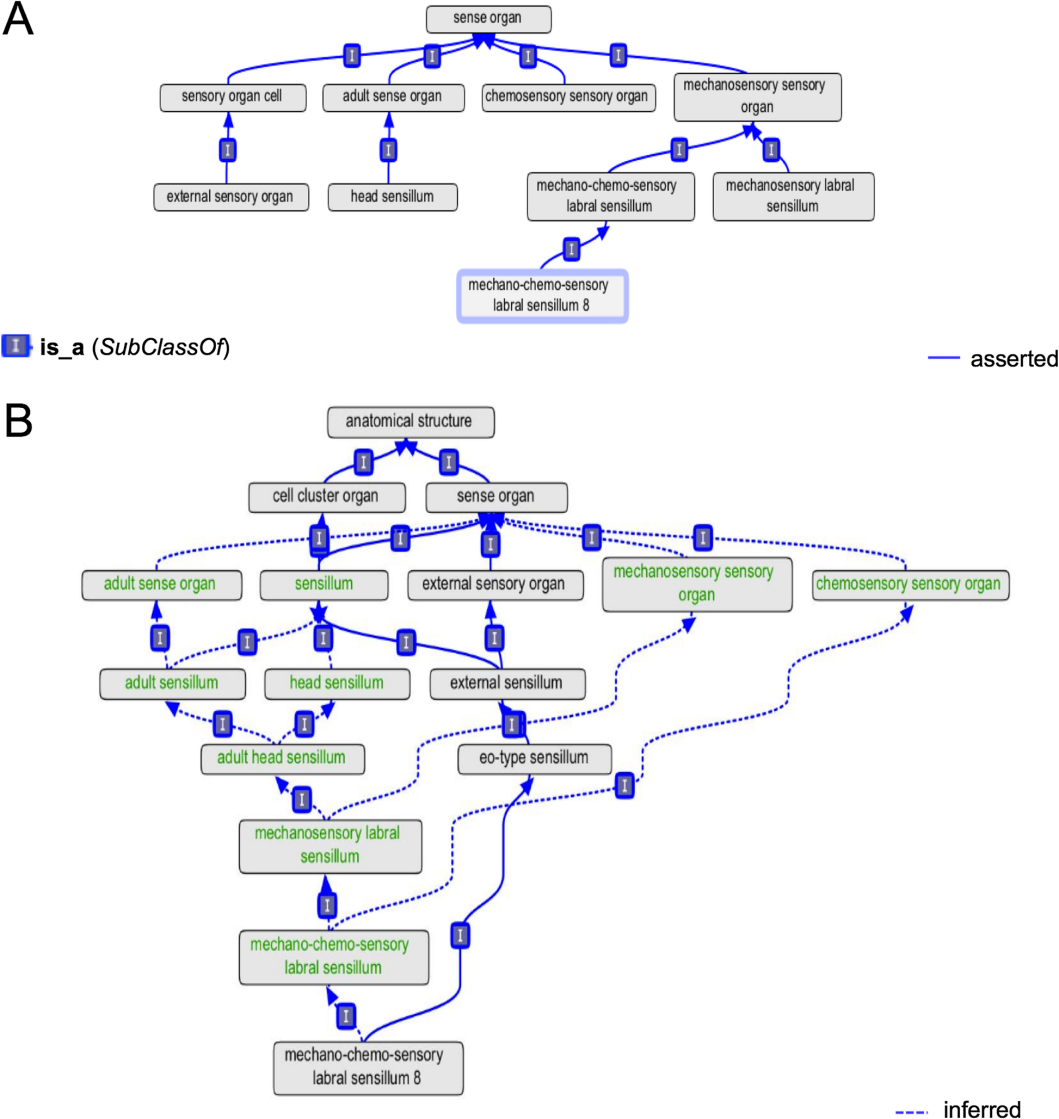
**Before and after refactoring.** An example of incomplete classification, fixed by refactoring. **(A)** shows the classification of ‘mechano-chemosensory labral sensillum 8’ prior to refactoring. Many valid classifications are missing. Note also the erroneous classification of ‘external sensory organ’ as a type of ‘sensory organ cell’. **(B)** shows autoclassification of the same class after refactoring. Terms with *equivalent class* definitions are shown in green.

This path of development eventually became unsustainable. By 2006, the ontology had over 6000 classes, only 4% of which were defined. Ontologies with multiple axes of classification are extremely hard to maintain by hand beyond a certain size [19]. Editors wanting to add a new class will have to create all the appropriate classifications manually, but in a large and growing ontology it can be very difficult to ascertain what classifications are available. Even where this is clear, it can be very difficult to judge the most appropriate place in each classification hierarchy to place the new class. This problem grows more difficult as the ontology grows, as more axes of classification are added and is compounded by the arrival of new editors lacking the tacit knowledge of those who created the ontology. In the absence of textual definitions, the tacit knowledge required for maintenance includes the meanings of the terms themselves and the reasons for existing classifications. For these reasons, the DAO classification hierarchy had accumulated gaps, redundancies, errors and duplications. (See Figure 1 for an example of gaps in the classification hierarchy).

A much more sustainable approach is to formally specify the properties of classes, where possible specifying a set of necessary and sufficient conditions for class membership. Standard OWL reasoning software can then be used to automate classification. Making the conditions for class membership explicit using OWL axioms also makes it possible to query the ontology for classes with particular combinations of properties. When combined with declarations of disjointness between classes (e.g. nothing can be both a muscle cell and a neuron) and other formalisms, a reasoner can also be used to detect errors.

We have transformed the DAO into a richly formalised ontology in OWL in which the majority of terms have textual definitions and much of the classification is automated. Here we describe its content, both formal and informal, its interconnection with other ontologies and its usage. Links for downloading various versions of the DAO and accessing documentation are provided in Table 1. The work described in this paper references release 2013-07-26 (see Table 1).

**Table 1:** Accessing the DAO

**Target**	**Base URL extension**
Homepage	fbbt
Term request tracker	fbbt/tracker
Pre-reasoned OBO version, no imports	fbbt/fbbt-simple.obo
Full OWL version withimports	fbbt/fbbt-non-classified.owl
Version described in thispaper	fbbt/releases/2013-07-26/
Full download guide	fbbt/downloads
Individual term details for FBbt_0000423 - Resolves programmatically to XML	FBbt_0000423

The DAO is openly available under a Creative Commons Attribution licence. DAO files, documentation and related resources can all be accessed via Persistent URLs (PURLs). The base URL for all PURLS is http://purl.obolibrary.org/obo/. Column 1 describes the targets of various PURLs specified in Column 2 as extensions to this base URL. The two columns should be combined without any additional spaces. e.g. http://purl.obolibrary.org/obo/fbbt/fbbt-simple.obo. We recommend opening OWL versions in Protégé 4.3 and classifying the full OWL version using the ELK reasoner [9]. The files in dated release folders follow the same naming convention as the latest versions.

## Results

### Criteria for inclusion in the DAO

One of the main aims of the DAO is to provide a queryable reference source for information about wild-type Drosophila anatomy. For this reason, we only include named classes where there is good scientific evidence for the presence in wild-type animals of structures with the properties described in both formal and informal components of the class definition^a^. Access to this evidence is provided by links to the relevant literature and sometimes in the form of free text summaries provided as a comment separate from the class definition (see the definition of *sound activated Johnston organ neuron* below). In order to conform to this inclusion criterion, we have occasionally obsoleted mistakenly added classes that refer to structures not present in wild-type *Drosophila*.

### Informal definitions

We have added well-referenced, textual definitions to over 73% of classes. This work was co-ordinated with formalisation and, in many cases, included the addition of comments containing a brief description of the evidence for assertions made in both formal an informal components of the definition. Formal assertions of properties are supported by references attached to the informal definition, allowing users and editors to quickly access the literature to judge for themselves whether the assertions are justified.

Textual definitions in the DAO follow approximately an Aristotelian genus and differentia pattern. The definitions first state a general classification or genus (e.g. ‘A neuron that...’) and then refine this with an account of the properties (differentia) that make members of this class different from others with the same classification. We optionally supplement this with a brief account of key properties that do not apply to all members where we consider such information useful to our users. References are included within the text of definitions, following typical academic style, as well as in the form of a list of identifiers to be used for rolling a hyperlinked bibliography for display.

For example: 

**name:** sound activated Johnston organ neuron

**definition:** A Johnston organ neuron (JON) that is activated by near-field sound ranging from 19 Hz to 952 Hz, maximally at 90 dB (Kamikouchi et al., 2009; Yorozu et al., 2009). These neurons are transiently (phasically) activated by the onset and offset of arista displacement. Cells preferentially activated by low-frequency vibration are loosely distributed as a ring in the middle layer of JON cell bodies. Higher frequencies preferentially activate JON neurons with cell bodies located mainly in the inner layer, directly surrounding the antennal nerve (Kamikouchi et al., 2006).

**comment:** Response to sound and arista displacement has been determined electrophysiologically and by using the calcium sensor GCaMP (Yorozu et al., 2009, Kamikouchi et al., 2009).

Wherever possible, we use consistent patterns to define groups of similar classes, such as neuron classes defined by lineage and innervation pattern, or muscle classes defined by their location, origin and insertion.

### Synonyms and disambiguation

Anatomical terminology varies between different research groups and over time. *Drosophila* anatomy has a long history, giving ample time for the development of varied and sometimes conflicting usage of terminology. The DAO would be of limited usage if it did not reflect this: there is no way to know *a priori* what terminology users will be familiar with; text miners need to be able to match the variety of terms they encounter in the literature with appropriate ontology terms.

To support textual searching and text mining, we have annotated many DAO terms with multiple synonyms. Wherever possible, these synonyms are linked to papers where they originate or that provide examples of their usage. For example, for the entire larval musculature, we have added referenced synonyms reflecting the two major nomenclatures currently in use [20,21] and a number of variants on them.

Where there are conflicting uses of terminology in the literature, we note this with a disambiguation comment. For example: 

**name:** ovariole

**comment:** The term ovariole is sometimes incorrectly used to refer to individual egg chambers. Please use the term ‘egg chamber’ for this.

### Refactoring the DAO

In order to improve the accuracy and completeness of classification in the DAO, we have refactored it to reduce asserted multiple inheritance classification. We have achieved this by adding formal specification of the properties of classes and then using this specification to infer a large proportion of the classification hierarchy. This work has been co-ordinated with the addition of textual definitions and references, with the references providing a link to the evidence for assertions in both formal and informal components of the definition. At the same time, we have added declarations of disjointness in order to provide basic error checking.

Fundamental declarations of disjointness hold between high-level anatomical classes. Adding these provides basic sanity checking for the ontology. For instance, nothing can be both a cell and a multicellular structure, or be both multicellular and acellular. As well as providing basic sanity checks, such fundamental declarations of disjointness are useful for detecting mistakes much further down the hierarchy. An error picked up early in refactoring was the misclassification of ‘external sensory organ’ (FBbt_00005168), a class of multicellular structure, as a type of ‘sensory organ cell’ (FBbt_00005163) (Figure 1).

Such checks can only work if there is sufficient classification in place. Prior to refactoring, only 240/6024 DAO terms were classified to root and 933 had no classification at all. In the current ontology all terms are classified using terms mapped to the Common Anatomy Reference Ontology (CARO) [22] or extensions to it (https://code.google.com/p/caro2/). CARO classes are treated as pairwise disjoint. Cells are all classified, via a separately maintained file of bridging axioms, using the Cell Ontology (CL) [17]. These basic classifications are a pre-requisite for much of the consistency checking and automated classification we have implemented and are also the basis for cross integration of anatomy ontologies by projects such as Uberon [18].

Rather than applying a rigid insistence on a single axis of classification, we have adopted a pragmatic approach, targeting easily formalisable classifications and tolerating dual inheritance where formalisations required to remove it were not obvious. Except for a few general class axioms used to specify disjointness, formalisation is restricted to the expressiveness of OBO format 1.4 [11] Much of the formalisation required new relations, which we developed in co-ordination with the OBO relations ontology and the cell ontology.

#### Patterns of formalisation

The main axes of classification we targeted for formalisation were partonomy and function. There are ample opportunities for leveraging the hierarchy of **part_of** (BFO_0000050) relationships to automate classification. For example we can formally specify the class *larval sensillum* (FBbt_00002782) as: 

‘larval abdominal sensillum’ *EquivalentTo* sensillum *that***part_of***some* ‘larval abdomen’

We specify function using **capable_of** (RO_0002215) [17] or **capable_of_part_of** (RO_0002216) relationships to terms from the biological process branch of the GO. These two relations are linked by the property chain: 

**capable_of***o***part_of***SubPropertyOf***capable_of_part_of**

**Figure 2:**
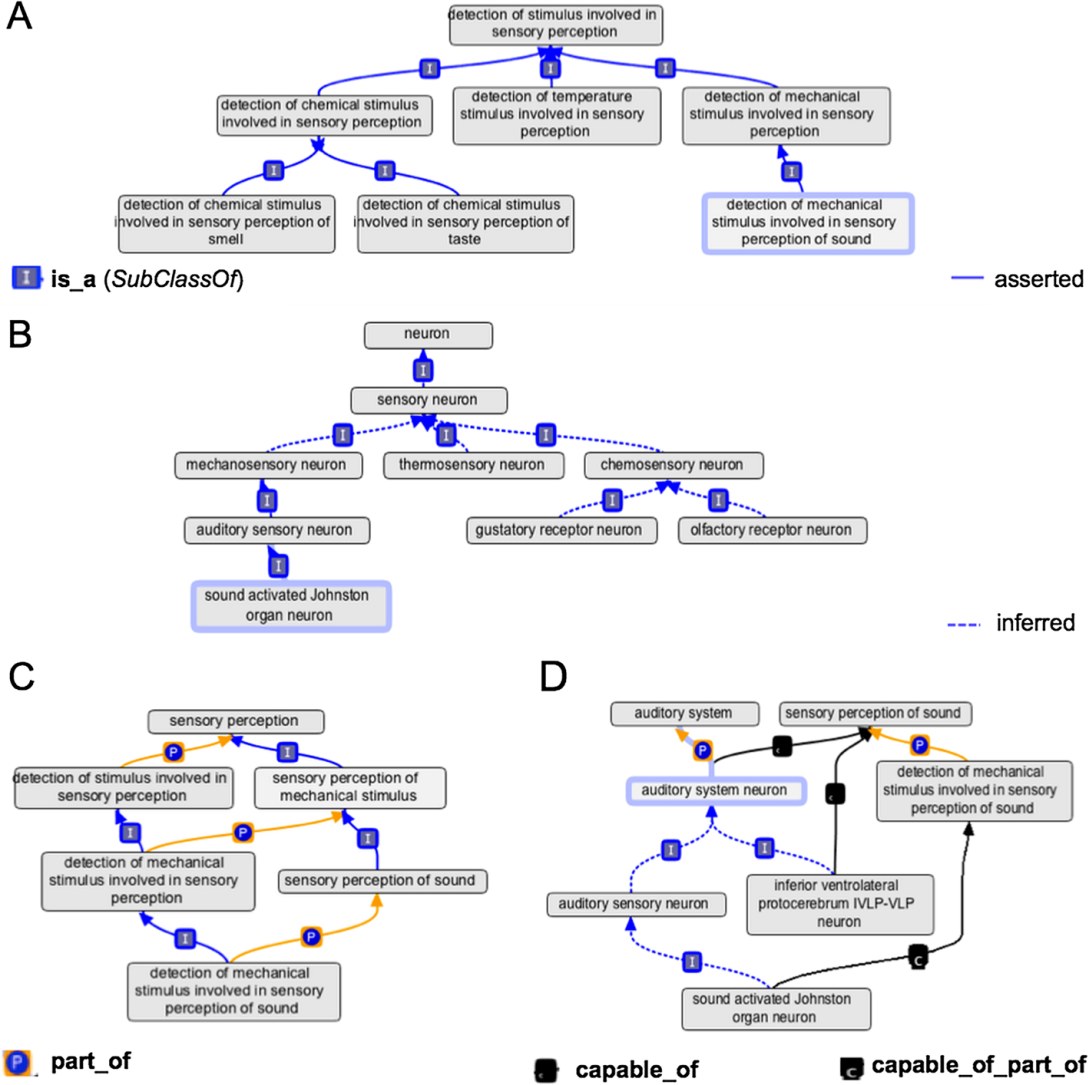
**Auto-classification of sensory modality.(A)** Classification under ‘detection of stimulus involved in sensory perception’. **(B)** Inferred classification of sensory neuron classes with sensory modality defined using the pattern: *EquivalentTo* neuron *that***capable_of***some* ‘detection of stimulus involved in sensory perception’ or one of its subclasses. **(C)** Classification and part relationships between subclasses of ‘sensory perception’ and subclasses of ‘detection of stimulus involved in sensory perception’. **(D)** Populating the auditory system. An auditory system neuron is defined as “any neuron that is **capable_of_part_of** (*some*) ‘sensory perception of sound”’. This property is directly asserted for ‘inferior ventrolateral protocerebrum IVLP-IVLP neuron’, and inferred for ‘auditory sensory neuron’, which is defined as “neuron *that***capable_of** (*some*) ‘detection of mechanical stimulus involved in sensory perception of sound”’. Inference comes from the part relation between the two GO terms and a property chain stating that if X **capable_of** Y and Y **part_of** Z then Z **capable_of_part_of** Z. All auditory system neurons are asserted to be **part_of** (*some*) ‘auditory system’, an assertion that is inherited by all classes inferred to be subclasses of ‘auditory system neuron’.

This can be read as stating the rule: If X **capable_of** Y and Y **part_of** Z then X **capable_of_part_of** Z. With this in place, we can leverage both the part and class hierarchies in the GO to structure the DAO (see Figure 2 for examples).

Much of the formalisation of the DAO follows well-documented design patterns (http://purl.obolibrary.org/obo/fbbt/doc/odp). Nested class expressions are not permitted in OBO 1.4, so design patterns typically refer to simple subject, relation, object relationship patterns with existential quantification (subject **relation***some* object). DAO design patterns have two components: (a) A specification of how to record a particular property (such as sensory modality) for some specified (subject) class of structure using a particular combination of relation and object class; (b) A set of high level classes defined using *EquivalentClass* axioms that automate classification, and in some cases partonomy, for classes that use the pattern defined in (a).

The formal definition and classification of nervous system components by their sensory modality provides a good example of this (see Figure 2). The GO biological process term *detection of stimulus involved in sensory perception* (GO_0050906) has a set of subclasses that are differentiated by the physical nature of the stimulus. Figure 2A shows a portion of this class hierarchy. A similar set of terms define the process of *sensory perception* (GO_0007600) with each detection of stimulus class standing in a **part_of** relationship to a sensory perception class defined by the same stimulus type (three such pairs are shown in Figure 2C).

The design pattern for specifying the sensory modality of sensory neurons is simply: 

*SubclassOf* <neuron or one of its subclasses>*that***capable_of***some* <‘detection of stimulus involved in sensory perception’ or one of its subclasses>

This is used as part of the formal definition of *sound activated Johnston organ neuron* (FBbt_00100002) (Figure 2B,D). A set of high level classes for sensory neurons, differentiated according to sensory modality, are defined for each ‘detection of stimulus’ class, following the pattern. 

‘sensory neuron’ *EquivalentTo* neuron *that***capable_of***some* <‘detection of stimulus involved in sensory perception’ or one of its subclasses>

A reasoner can then automatically classify neurons whose definition follows the design pattern under the appropriate general class. For example, Figure 2B shows the inferred classification of *sound activated Johnston organ neuron*.

The design pattern for asserting a downstream function for a neuron in sensory perception is: 

*SubclassOf* <neuron or one of its subclasses>*that***capable_of_part_of** <‘detection of stimulus involved in sensory perception’ or one of its subclasses>

This is used as part of the formal definition of *inferior ventrolateral protocerebrum IVLP-VLP neuron* (FBbt_00110126) (Figure 2D). For each sensory system, we define a general class using this pattern, and assert that all members of this class are part of the relevant sensory system. For example, for the auditory system we define: 

‘auditory system neuron’

*EquivalentTo* neuron *that***capable_of_part_of***some* ‘sensory perception of sound’

*SubclassOf***part_of***some* ‘auditory system’^b^

Figure 2D shows the automated classification that results from these formalisations, including automated population of the partonomy of the auditory system (see figure legend for details). Over 1400 classes of neuron and sense organ in DAO are automatically classified according to their sensory modality using this pattern. Many other design patterns are used in the DAO. These include patterns for representing neuro-anatomy [6], classifying larval musculature and classifying larval trachea. In total, almost 50% of the > 10,000 classifications in the current, pre-reasoned versions of the DAO originate as inferred classifications.

#### Using images to define ontology terms

**Figure 3:**
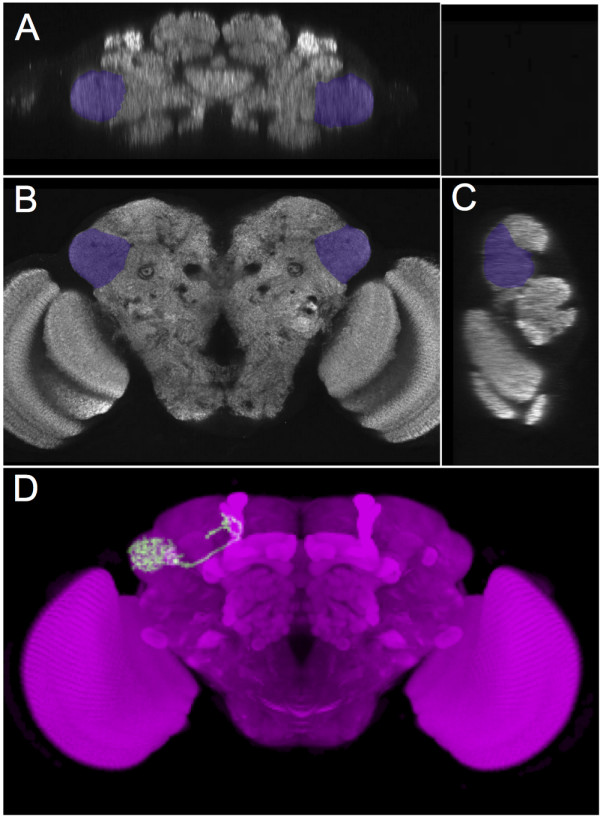
**Defining with images.(A)** horizontal **(B)** frontal and **(C)** sagital sections through a standard reference *Drosophila* brain in which the lateral horn region is highlighted in purple with boundaries defined by the BrainName standard^c^. We record a formal connection between an OWL individual representing the painted region and the class as: ‘lateral horn’ *EquivalentTo***has_reference_image***value* ‘BrainName exemplar lateral horn’. **(D)** shows an image of single neuron that has been registered to the standard brain. Image analysis has determined overlap between the neuron and the region defined as lateral horn in the standard. Based on this, the neuron in the image has been annotated with the axiom: *SubClassOf***overlaps***some* ‘lateral horn’. (Painting of standard brain by A.Jennet and K.Shinomiya. The single neuron image in panel C was derived from a neuron imaged by FlyCircuit [23] with registration and image analysis by MC and Gregory SXE Jefferis).

Anatomy is an intensely visual subject. Anatomical images can communicate the meaning of anatomical terms much more rapidly and efficiently than text and can be irreplaceable as a means of communicating the position of boundaries. Textual definitions can be enhanced by providing example images and schematic drawings - either informally annotated (FlyBase provides over 1100 of these for the DAO), or annotated as OWL individuals (VFB provides over 17000 3D images of neurons and neural clones, annotated with OWL axioms referencing DAO classes). But it can also be useful to explicitly define an anatomical class in relation to a standard reference image. This is particularly useful where there is a standard 3D reference image available that defines the boundaries of regions and a standard co-ordinate space onto which multiple images can be mapped via registration (warping) algorithms. There are two such standards available for the adult *Drosophila* brain, FlyCircuit [23] and BrainName^c^. We have defined OWL individuals corresponding to each of the painted brain regions in the BrainName standard brain. Part of the formal definition of each term in the DAO for one of these brain regions is a formal link to the individual corresponding to the appropriate region of the standard brain. Figure 3A-C shows one such brain region, the lateral horn, and the axiom that links this individual brain region to the class ‘lateral horn’ in the ontology.

The VFB project has generated 10’s of thousand of OWL axioms recording the overlap of individual registered neurons to brain regions defined in this way. Figure 3D shows a neuron, registered to the standard brain, that overlaps the painted region defining the boundaries of the lateral horn (see figure legend for formalisation details).

### Current content

**Figure 4:**
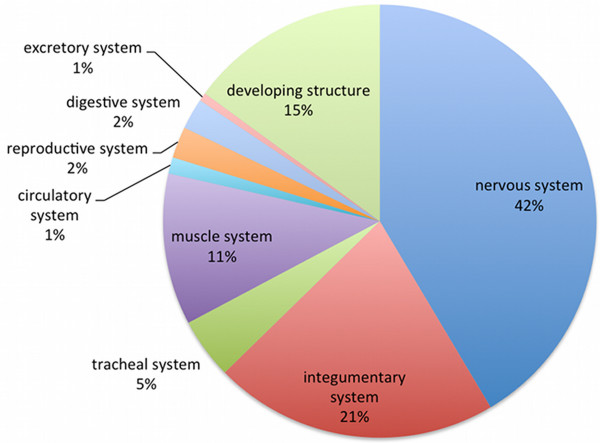
**DAO content by system.** Content of the DAO, divided by anatomical system.

As a result of the work described here, the DAO now consists of over 8000 classes and over 16000 logical axioms covering all aspects of *Drosophila* anatomy from the start of development through to mature adulthood. Through the work of the VFB project, neuro-anatomical content is particularly rich. Figure 4 shows the proportion of terms by system. 73% of classes have textual definitions, which in total reference almost 500 publications. As a result of refactoring, approximately half of over 10,000 classifications in the pre-reasoned version of the ontology are now inferred rather than asserted. Extensive use of disjointness axioms provides error checking.

### Applications

#### Semi-formalised annotation of phenotype and expression using DAO

The majority of annotation with the DAO records phenotypes or expression patterns and consists of associations between DAO terms, genetic features and terms from other ontologies stored in a relational database. While there is generally some level of informal agreement about the meaning of these associations, their formal semantics remain unspecified. This approach is used by FlyProt (http://www.flyprot.org/) and RedFly [24] and most extensively by FlyBase [3], which uses it in over 260,000 annotations of phenotypes and expression patterns and to annotate over 1100 anatomical images. These resources consume a simple, pre-reasoned version of the ontology in OBO format (see Table 1), which they use to group annotations via combined class and part hierarchies.

#### Formal annotation using DAO

An alternative approach is to formalise annotations assertions in an OWL knowledge base. This allows the semantics of annotation to be more precisely specified and allows annotations to be classified and queried using an OWL reasoner. The resulting queries have more precisely specified semantics than is typical for SQL queries of annotations and the results lists are enriched via logical inferences that would not be available via SQL.

VFB uses this approach to annotate 3D images of neuroanatomical structures. It currently has over 17000 images of neurons, neuron clusters and clones annotated as OWL individuals. These are all typed using named classes and class expressions referencing the DAO. Typing uses a mixture of manual annotation and automated annotation based on computation of voxel overlap to reference regions in a standard brain to which all images are registered. Combining the resulting knowledge-base with the DAO and classifying using an OWL reasoner, auto-classifies many of neurons and integrates them into the VFB query system.

### The DAO as a queryable reference for *Drosophila* anatomy

So far, VFB is the only resource to use the DAO as a *queryable* reference for *Drosophila* anatomy. While VFB only provides access to neuroanatomical content of the DAO, it displays a complete set of associated information for all terms it displays, including a definition, comments and synonyms along with hyperlinked references, classifications and relationships. It also provides a set of queries of anatomy, images and annotations, tailored to the class of term being displayed. For example, users can run queries from brain region terms to find all the neuron classes that have synaptic terminals in that region. Both the choice of queries available for any given term and the queries themselves are specified using OWL-DL queries, run using the OWL reasoner ELK [9]. Annotations of expression and phenotype are pulled from FlyBase but use a pre-query in OWL that finds significantly more classes for grouping annotations than simple grouping via class and part hierarchies [5]. Queries for images use the DAO in combination with an OWL knowledge base.

### Support for using the DAO in annotation

We maintain a google code site (https://code.google.com/p/vfb-annotation-tools/) dedicated to templates for using the spreadsheet based annotation system, Populous [25] to annotate using the DAO. We chose Populous over alternatives such as Phenote (http://www.phenote.org/) because a spreadsheet-based annotation system has almost no overhead for non-expert users and because of its support for logically defining both input terms and output annotations in OWL. The resulting spreadsheets have a validation system, ensuring that users only annotate with valid term names. IDs are linked behind the scenes so that users do not need to track them. This can be used for simple informal tagging systems. It also provides the option of writing the resulting spreadsheets to OWL using templates specified via OPPL [26]. Doing so allows the semantics of annotation to be strictly specified and annotations to be queried in combination with the DAO using OWL-DL queries.

### Future directions

There remains significant scope for useful refactoring of the DAO. 27% of classes remain undefined, with significant work remaining to complete definitions for the adult musculature and embryonic developmental anatomy. A number of classes still have only crude classifications, including over 40 only classified as ‘anatomical entity’. Refining the classification of crudely classified classes is a current priority, along with adding disjointness axioms to improve error checking.

Other future work is likely to focus on improving the representation of stage and its relationship to partonomy. There are no formal relationships between terms in the DAO and those in the *Drosophila* Stage Ontology. These would be useful for a number of purposes - not least in formalising the definition of phenotype and expression curation in FlyBase that combines these two ontologies.

The DAO currently uses non-rigid classes [27] for structures that persist between life stages. For example, we have the general term *mushroom body* (FBbt_00005801) with stage-specific subclasses defined using part relationships to terms for the whole animal during some life-cycle stage: 

‘adult mushroom body’ *EquivalentTo* ‘mushroom body’ *that***part_of***some* adult

A major problem with this approach is that it requires different partonomies for different stages, with continuity of classes of structure between different stages being represented using developmental relations (continuity of individuals between non-rigid classifications can not be represented in OWL). This causes problems for recording partonomy in anatomy that changes rapidly - as is the case with much developmental anatomy. Recent work on temporal indexing of part relations as part of work on version 2.0 of the Basic Formal Ontology (BFO) may provide at least partial solutions to this problem.

## Conclusions

The addition of referenced textual definitions for over 73% of terms in the DAO, along with extensive refactoring to check for errors and infer multiple inheritance classification has dramatically improved the accuracy and usefulness of the DAO as a queryable reference for wild-type anatomy. This is well illustrated by its usage on the VFB site as a source of information about *Drosophila* neuroanatomy and as a driver of anatomical queries. The addition of textual definitions has made it much easier for curators to rapidly find appropriate terms for annotation with the DAO, while refactoring has dramatically improved the accuracy and completeness with which those annotations are grouped on FlyBase and other projects hosting DAO-based annotation. The VFB project again illustrates this well: prior to this work, the accuracy and completeness of the classification hierarchy for *Drosophila* neuro-anatomy was simply too poor to have been useful for driving VFB.

Prior to refactoring, editing the ontology by hand was error prone and tended to result in incomplete classifications. Refactoring, along with the documentation of design patterns, has put future development of the ontology on a sustainable footing. The patterns of formalisation used in the DAO are likely to be useful to developers of other anatomy ontologies.

The extensive use of terms from external ontologies in refactoring provides a potential basis for cross-species querying based, for example, on shared function or cell types. The links to external ontologies have already been used to experimentally incorporate the DAO into an extended version of the Uberon multi-species anatomy ontology [18].

## Methods

Please see Table 1 for details of how to access ontology versions and documentation. This paper refers to the versions found in release 2013-07-26.

The master version of the DAO has remained in OBO format throughout refactoring and formalisation. During editing, the ontology was continuously converted to OWL and classified to test inference and consistency. We used Protégé 4 along with the ELK reasoner to browse inferred classification and run test queries. We also used a continuous integration server (Jenkins) to add automated definitions, check syntax and consistency, and generate various flavours of derivative OBO and OWL following every commit to our version control respository.

For every term from an external ontology used in a DAO axiom we import all terms and axioms on paths to root from a pre-reasoned version of the external ontology using OORT (https://code.google.com/p/owltools/wiki/OortIntro).

Conventions used in this paper: classes are referred to in free text using their label italics, followed by their OWL short form ID in brackets. Following the OBO foundry ID standard (http://www.obofoundry.org/id-policy.shtml) a full URI can be generated by prepending http://purl.obolibrary.org/obo/. In most cases this will URI will resolve to OntoBee (http://www.ontobee.org/), returning XML if accessed programatically. OBO IDs can be derived by converting the underscore in a short-form ID to a colon. All formal axioms are expressed in OWL Manchester syntax (OWL-MS) (http://www.w3.org/TR/owl2-manchester-syntax/). OWL-MS keywords are italicised. Note that *that* and *and* are interchangeable in OWL-MS. We choose which to use based entirely on readability. Object properties (relations) are in bold. The names of OWL entities (e.g. classes, object properties) are quoted only if they contain spaces.

## Endnotes

^a^ We acknowledge that this aim is likely to be imperfectly realised in many cases, but our goal is simply an acceptably accurate reference source.

^b^ This combination of equivalent class and subclass axioms constitutes a hidden general class inclusion axiom (GCI). It is expressed in this way, rather than as a separate GCI, for compliance with OBO format.

^c^ Ito K, Shinomiya K, Armstrong J, Boyan G, Hartenstein S V Harzsch, Heisenberg M, Homberg U, Jenett A, Keshishian H, L R, Rossler W, Simpson J, Strausfeld N, Strauss R, Vosshall L: **A Coordinated Nomenclature System for the Insect Brain.** Submitted

## Competing interests

The authors declare that they have no competing interests.

## Authors’ contributions

GG contributed to early efforts to add textual definitions and made extensive links between anatomy terms and images for FlyBase. DOS took over as editor in 2006 and remained senior editor of this ontology to September 2013. He has been responsible for the formalisation strategy and much of its implementation and for development of infrastructure and tools used in DAO development. SR was a DAO editor from October 2009 to August 2011 during which time he contributed a large number of textual definitions. MC has been an editor since September 2011 and has made very significant contributions to definitions and formal content relating to the nervous system. This paper was largely written by DOS, with edits and suggestions provided by other authors. All authors read and approved the final manuscript.

## References

[B1] Sprague J Bayraktaroglu L Bradford Y Conlin T Dunn N Fashena D Frazer K Haendel M Howe DG Knight J Mani P Moxon SA Pich C Ramachandran S Schaper K Segerdell E Shao X Singer A Song P Sprunger B Van Slyke CE Westerfield M The Zebrafish Information Network: the zebrafish model organism database provides expanded support for genotypes and phenotypes Nucleic Acids Res 2008 4 Database issue D768 D772 17991680 10.1093/nar/gkm956 PMC2238839

[B2] Bult CJ Eppig JT Blake JA Kadin JA Richardson JE Airey MT Anagnostopoulos A Babiuk R Baldarelli RM Beal JS Bello SM Butler NE Campbell J Corbani LE Dene H Drabkin HR Forthofer KL Giannatto SL Knowlton M Lewis JR McAndrews M McClatchy S Miers DS Ni L Onda H Ormsby JE Recla JM Reed DJ Richards-Smith B Shaw DR The mouse genome database: genotypes, phenotypes, and models of human disease Nucleic Acids Res 2013 4 Database issue D885 D891 23175610 10.1093/nar/gks1115 PMC3531104

[B3] Grumbling G Strelets V FlyBase: anatomical data, images and queries Nucleic Acids Res 2006 4 Database issue D484 D488 16381917 10.1093/nar/gkj068 PMC1347431

[B4] Engel SR Balakrishnan R Binkley G Christie KR Costanzo MC Dwight SS Fisk DG Hirschman JE Hitz BC Hong EL Krieger CJ Livstone MS Miyasato SR Nash R Oughtred R Park J Skrzypek MS Weng S Wong ED Dolinski K Botstein D Cherry JM Saccharomyces Genome Database provides mutant phenotype data Nucleic Acids Res 2010 4 Database issue D433 D436 19906697 10.1093/nar/gkp917 PMC2808950

[B5] Milyaev N Osumi-Sutherland D Reeve S Burton N Baldock RA Armstrong JD The Virtual Fly Brain browser and query interface Bioinformatics 2012 4 3 411 415 10.1093/bioinformatics/btr677 22180411

[B6] Osumi-Sutherland D Reeve S Mungall CJ Neuhaus F Ruttenberg A Jefferis GS Armstrong JD A strategy for building neuroanatomy ontologies Bioinformatics 2012 4 9 1262 1269 10.1093/bioinformatics/bts113 22402613

[B7] OWL 2 Web Ontology Language Primer (Second Edition) - W3C Recommendation 11 December 2012 [http://www.w3.org/TR/owl2-primer/]

[B8] OWL 2 EL profile - W3C Recommendation 11 December 2012 [http://www.w3.org/TR/owl2-profiles/#OWL_2_EL]

[B9] Kazakov Y Krötzsch M Simančík F ELK reasoner: Architecture and evaluation. (From Proceedings of the 1st International OWL Reasoner Evaluation Workshop) CEUR Workshop Proc 2012 4 10

[B10] Golbreich C Horrocks I The OBO to OWL mapping, GO in OWL 1.1! (From Proc. of the third OWL Experiences and directions workshop) CEUR Workshop Proc 2007 4 1 9

[B11] Mungall C Ruttenberg R Horrocks I Osumi-Sutherland D Antezana E Balhoff J Courtot M Dietze H Day-Richter J Horridge H Ireland A Lewis S Manzoor S Hamid Tirmizi S OBO Flat File Format 1.4 Syntax and Semantics [http://oboformat.googlecode.com/svn/trunk/doc/obo-syntax.html]

[B12] Mungall CJ Bada M Berardini TZ Deegan J Ireland A Harris MA Hill DP Lomax J Cross-product extensions of the Gene Ontology J Biomed Inform 2011 4 80 86 10.1016/j.jbi.2010.02.002 20152934 PMC2910209

[B13] Deegan nee Clark JI Dimmer EC Mungall CJ Formalization of taxon-based constraints to detect inconsistencies in annotation and ontology development BMC Bioinformatics 2010 4 530 10.1186/1471-2105-11-530 20973947 PMC3098089

[B14] Gkoutos GV Hoehndorf R Ontology-based cross-species integration and analysis of Saccharomyces cerevisiae phenotypes J Biomed Semantics 2012 4 Suppl 2 S6 10.1186/2041-1480-3-S2-S6 23046642 PMC3448529

[B15] Gkoutos GV Mungall C Dolken S Ashburner M Lewis S Hancock J Schofield P Kohler S Robinson PN Entity/quality-based logical definitions for the human skeletal phenome using PATO Conf Proc IEEE Eng Med Biol Soc 2009 4 7069 7072 19964203 10.1109/IEMBS.2009.5333362 PMC3398700

[B16] Gkoutos GV Green EC Mallon AM Blake A Greenaway S Hancock JM Davidson D Ontologies for the description of mouse phenotypes Comp Funct Genomics 2004 4 6–7 545 551 18629136 10.1002/cfg.430 PMC2447424

[B17] Meehan TF Masci AM Abdulla A Cowell LG Blake JA Mungall CJ Diehl AD Logical development of the cell ontology BMC Bioinformatics 2011 4 6 10.1186/1471-2105-12-6 21208450 PMC3024222

[B18] Mungall CJ Torniai C Gkoutos GV Lewis SE Haendel MA Uberon, an integrative multi-species anatomy ontology Genome Biol 2012 4 R5 10.1186/gb-2012-13-1-r5 22293552 PMC3334586

[B19] Rector AL Modularisation of domain ontologies implemented in description logics and related formalisms including OWL Proceedings of the 2nd International Conference on Knowledge Capture 2003 New York: ACM 121 128 [http://doi.acm.org/10.1145/945645.945664]

[B20] Crossley AC Ashburner M, Wright TRF The morphology and development of the Drosophila muscular system The Genetics and Biology of Drosophila, Volume 2d 1980 London: Academic Press 499 560

[B21] Bate M Bate M, Martinez Arias A The mesoderm and its derivatives. In The development of Drosophila melanogaster The development of Drosophila melanogaster, Volume 2 1993 Cold Spring Harbor: Cold Spring Harbor Laboratory Press 1013 1090

[B22] Haendel MA Neuhaus F Osumi-Sutherland D Mabee PM Mejino JL Mungall CJ Smith B CARO–the common anatomy reference ontology. (in Anatomy Ontologies for Bioinformatics) Comput Biol 2008 4 327 349 Edited by Burger A, Davidson D, Baldock, R. Springer 10.1007/978-1-84628-885-2_16

[B23] Chiang AS Lin CY Chuang CC Chang HM Hsieh CH Yeh CW Shih CT Wu JJ Wang GT Chen YC Wu CC Chen GY Ching YT Lee PC Lin CY Lin HH Wu CC Hsu HW Huang YA Chen JY Chiang HJ Lu CF Ni RF Yeh CY Hwang JK Three-dimensional reconstruction of brain-wide wiring networks in Drosophila at single-cell resolution Curr Biol 2011 4 1 11 21129968 10.1016/j.cub.2010.11.056

[B24] Gallo SM Gerrard DT Miner D Simich M Des Soye B Bergman CM Halfon MS REDfly v3.0: toward a comprehensive database of transcriptional regulatory elements in Drosophila Nucleic Acids Res 2011 4 Database issue D118 D123 20965965 10.1093/nar/gkq999 PMC3013816

[B25] Jupp S Horridge M Iannone L Klein J Owen S Schanstra J Wolstencroft K Stevens R Populous: a tool for building OWL ontologies from templates BMC Bioinformatics 2012 4 Suppl 1 S5 22373396 10.1186/1471-2105-13-S1-S5 PMC3471341

[B26] Egaña M Rector A Stevens R Antezana E Applying ontology design patterns in bio-ontologies LNCS 2008 4 7 16

[B27] Guarino N Welty C Evaluating ontological decisions with OntoClean Commun ACM 2002 4 2 61 65

